# Supplementary material for: Triarylpyridine Compounds and Chloroquine Act in Concert to Trigger Lysosomal Membrane Permeabilization and Cell Death in Cancer Cells
Source: Cancers (Basel). 2020 Jun 18;12(6):1621. doi: 10.3390/cancers12061621 (PMC7352983; doi:10.3390/cancers12061621)
Supplement: Supplementary file 1 [file cancers-12-01621-s001.pdf]

Supplementary Files

Triarylpyridine Compounds and Chloroquine Act in Concert to Trigger Lysosomal Membrane Permeabilization and Cell Death in Cancer Cells

Jennifer Beauvarlet, Rabindra Nath Das, Karla Alvarez-Valadez, Isabelle Martins, Alexandra Muller, Elodie Darbo, Elodie Richard, Pierre Soubeyran, Guido Kroemer, Jean Guillon, Jean-Louis Mergny and Mojgan Djavaheri-Mergny

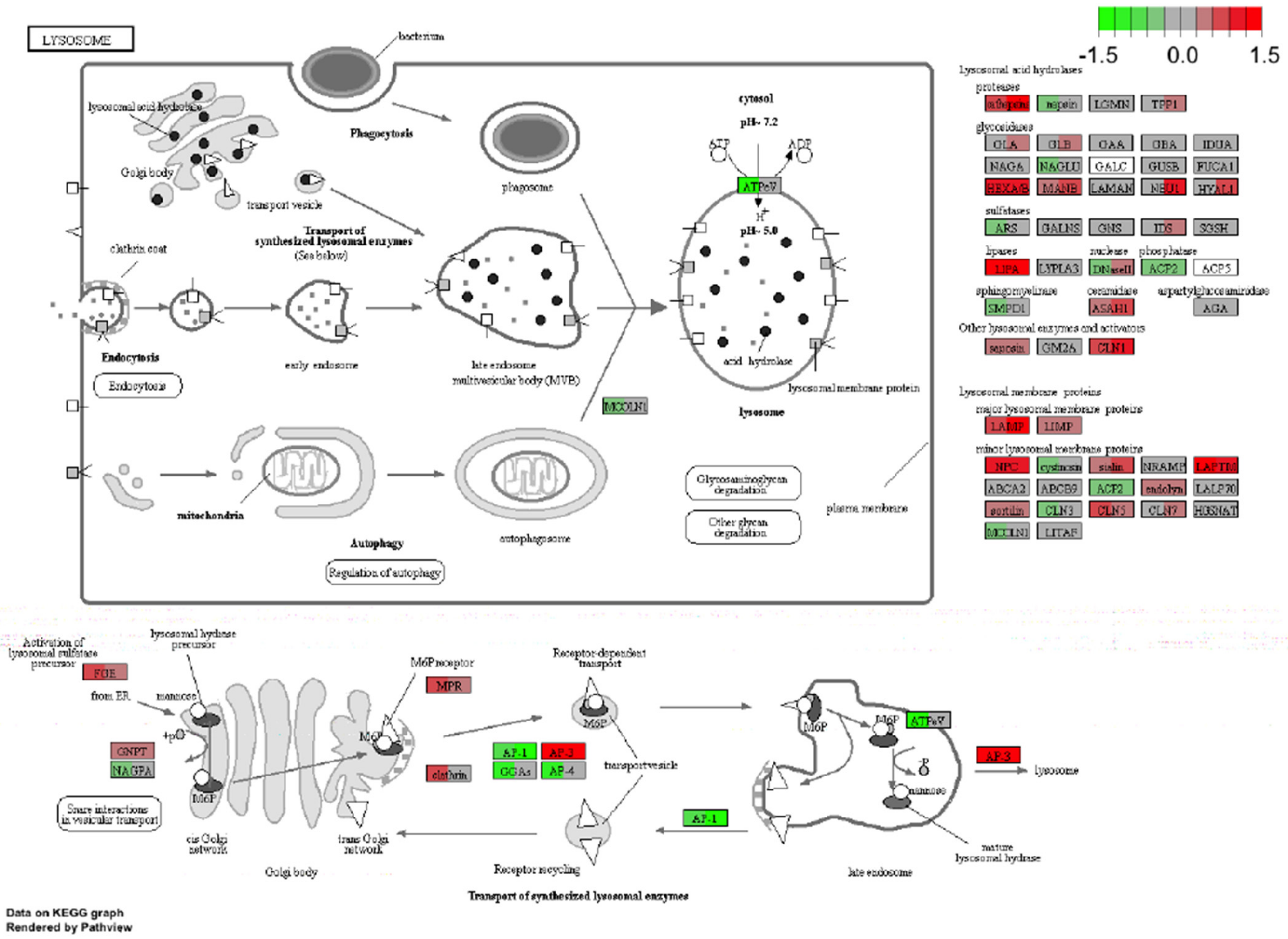

Figure S1. KEGG database depicting the lysosomal network alterations by 20A. HeLa cells were treated with 6 µM 20A for either 6 or 16 hours and then subjected to thetranscriptome analysis. Schematic representation of the lysosomal pathway from KEGG database (lysosome genome.jp) is shown. For each gene, the log fold changes 20A treated cells versus untreated cells is represented and denoted with a color code (from green (low) to red (high) shown in the top of the figure. The transcriptomic data after 6 hand 16 h treatment are presented in the left and right panel of each rectangle,respectively. A white box means that the transcript was not identified in the experiment.

A

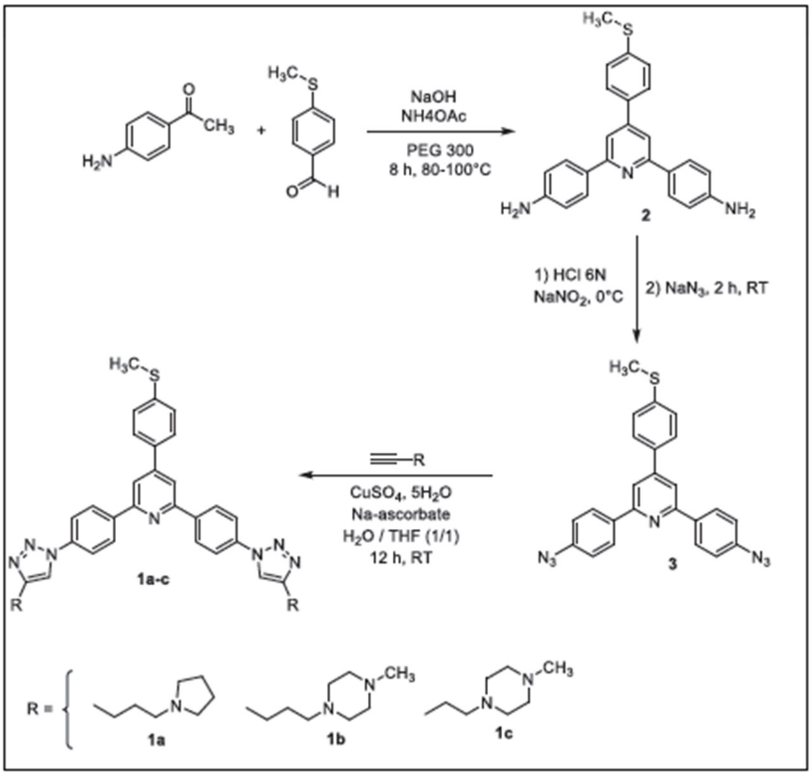

B

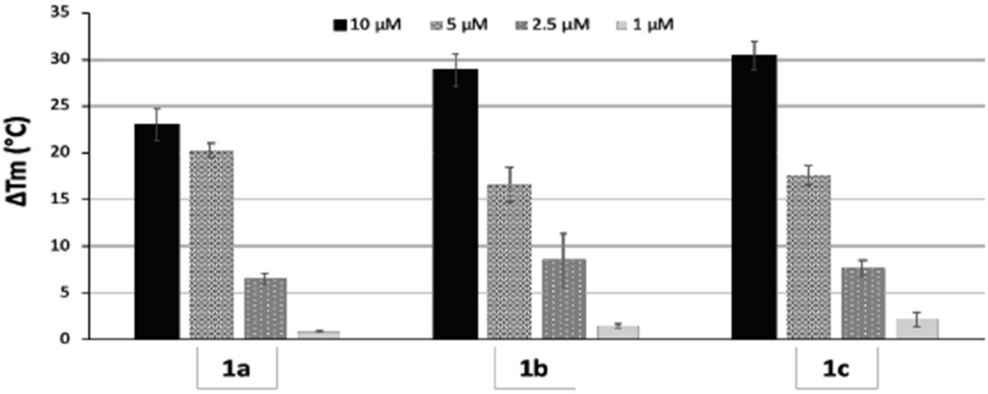

Figure S2. (**A**) Scheme for synthesis of 20A derivatives. A series of novel bis-triazole 2,4,6-triarylpyridines 1a-c have been synthesized through a.three steps procedure (Figure 1). The synthetic route involves the base-catalysed condensation of commercially available p-aminoacetophenone with p-thiomethyl benzaldehyde in PEG300 resulting the formation of 2,4,6- triarylpyridine 2.The di-amino 2 is then converted to corresponding diazido derivative 3 which is then subjected for coppercatalyzed azide-alkyne 1,3-dipolar cycloaddition reaction with various amine-terminal alkynes to afford 1,2,3-triazole derivatives 1a–c. (**B**) The 20A-derivatives (1a, 1b and 1c) stabilize an intramolecular quadruplex. F21T is. a dual labeled oligonucleotide able to fold into an intramolecular quadruplex. Increasing concentrations of each ligand (1–10 μM) led to an increase in Tm (in ° C) measured by FRET.

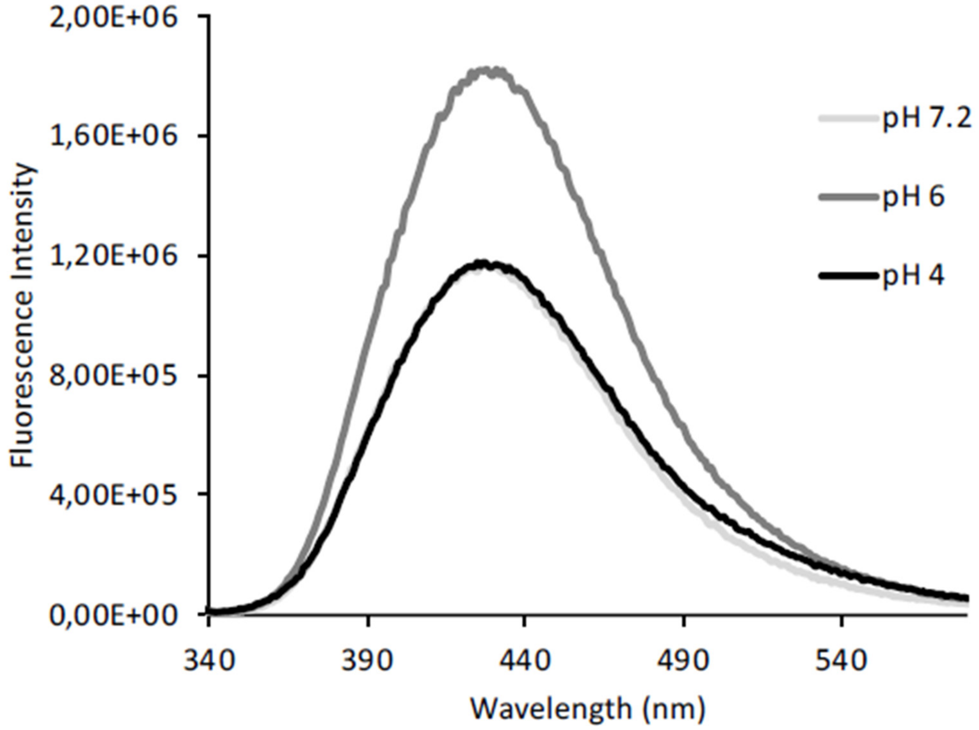

Figure S3. Fluorescence emission spectra of 20A compound at 3 different pH. The fluorescence spectra were recorded on a Fluoromax-4 Horiba spectrofluorimeter using 5 μM 20A concentration in 100 mM KCl, 10 mM Tris buffer at 25 °C with a quartz cuvette of 1 cm path length. Excitation was set at 300 nm.

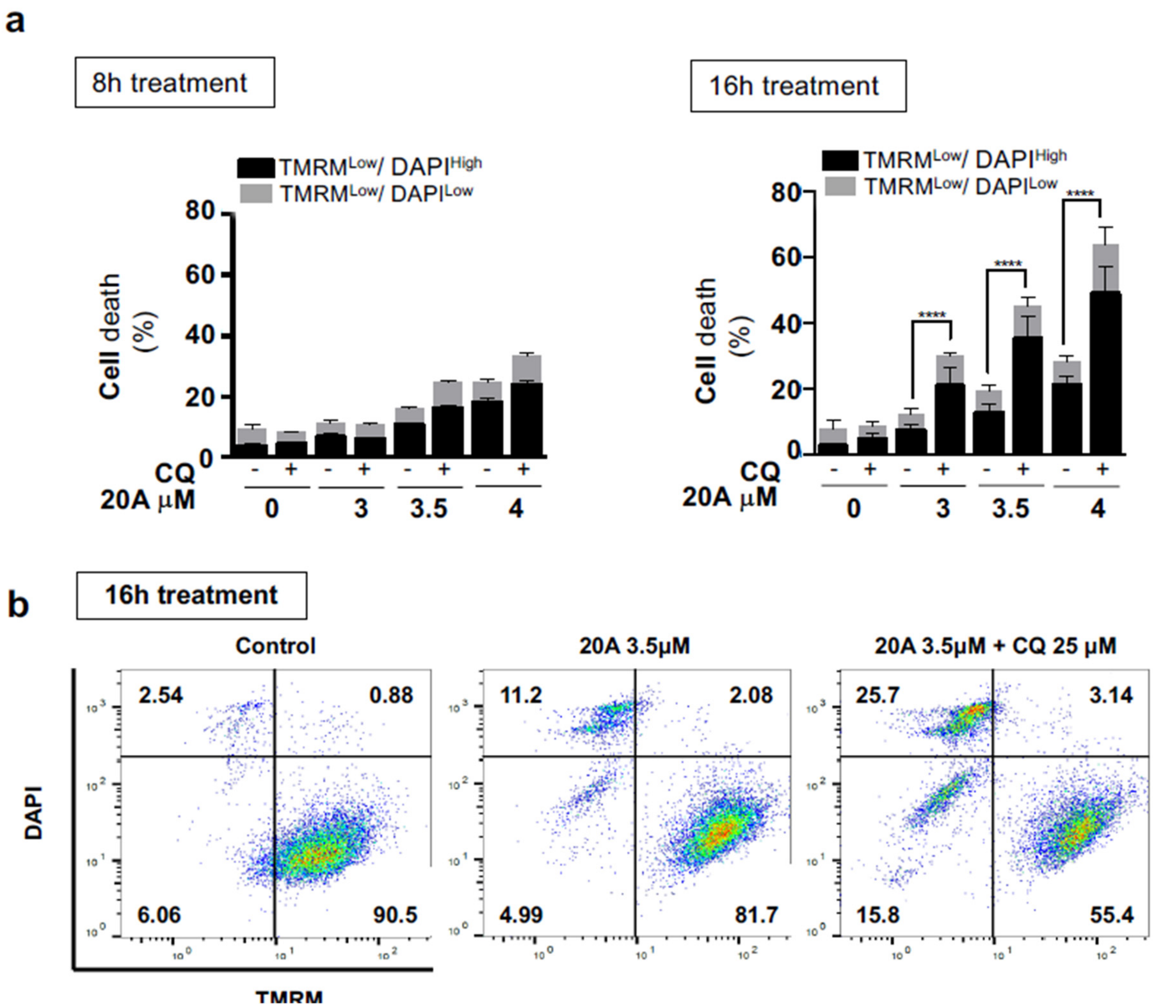

Figure S4. Evaluation of early and late apoptosis in U2OS cells treated by 20A plus chloroquine. (**a**) U20S cells were treated with the indicated concentration of 20A with or without 25 µM of chloroquine for either 8 h or 16 h. Cells were stained with 150 nM tetramethylrhodamine methyl ester (TMRM, from Molecular Probes-Life Technologies) and 5 µg/mL 4′,6-diaminidino-2-phenylindole (DAPI, from Molecular Probes-Life Technologies) for 30 min at 37 °C. Cytofluorometric determinations were carried out on a MACSQuant cytometer (Miltenyi Biotec, Bergisch Gladbach, Germany). Cell death was evaluated by scoring the percentage of TMRMLow/ DAPILow (early apoptosis) and TMRMLow/ DAPIHigh (late apoptosis) cells following flow cytometer analysis. **b)** Representative images of dot plots are shown for 16 hours of treatment with 20A ± chloroquine.

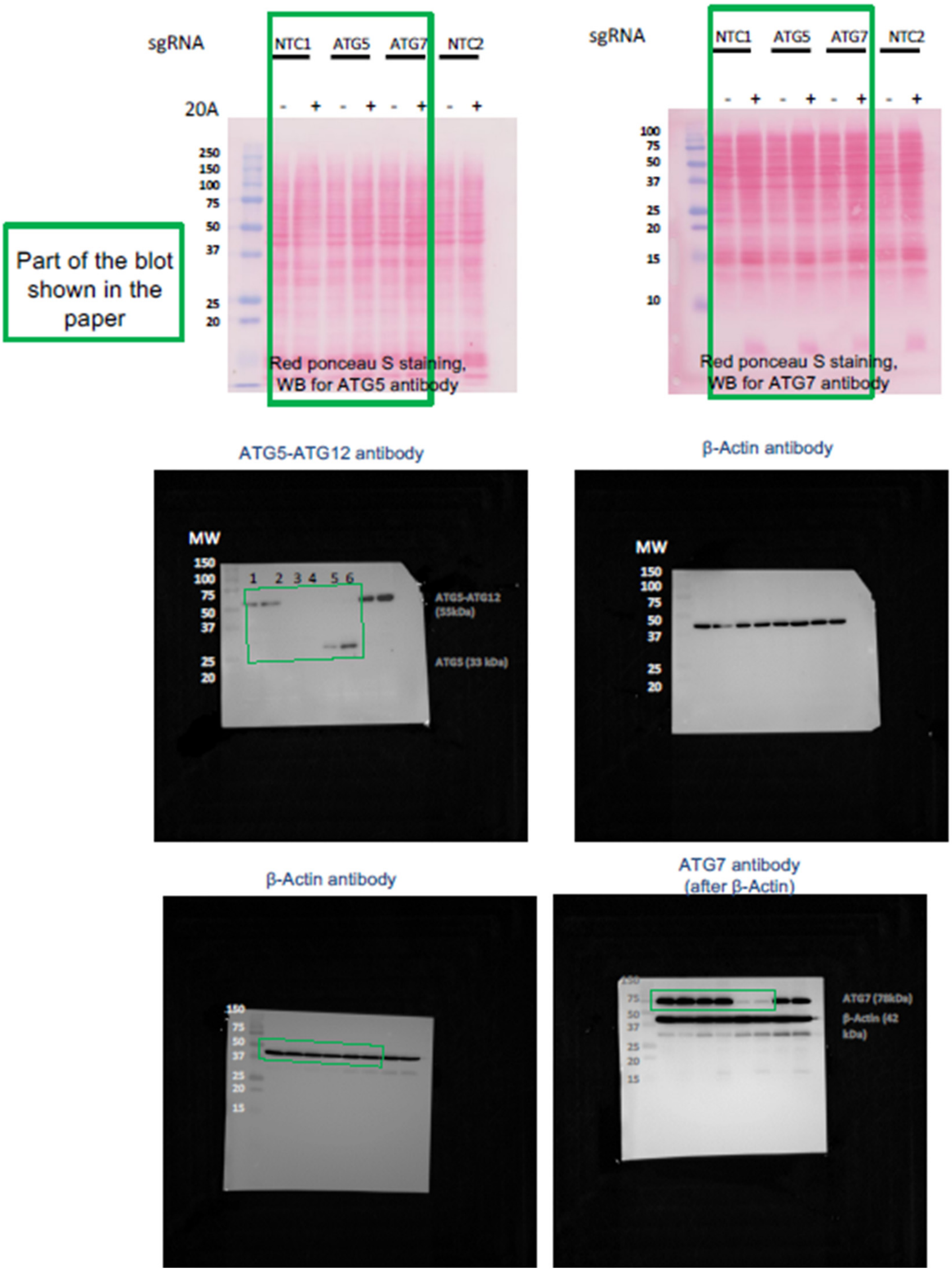

Figure S5. Full Western blot images for Western blotting experiments presented in Figure 3. Densitometry analyses are presented in Figure 3.

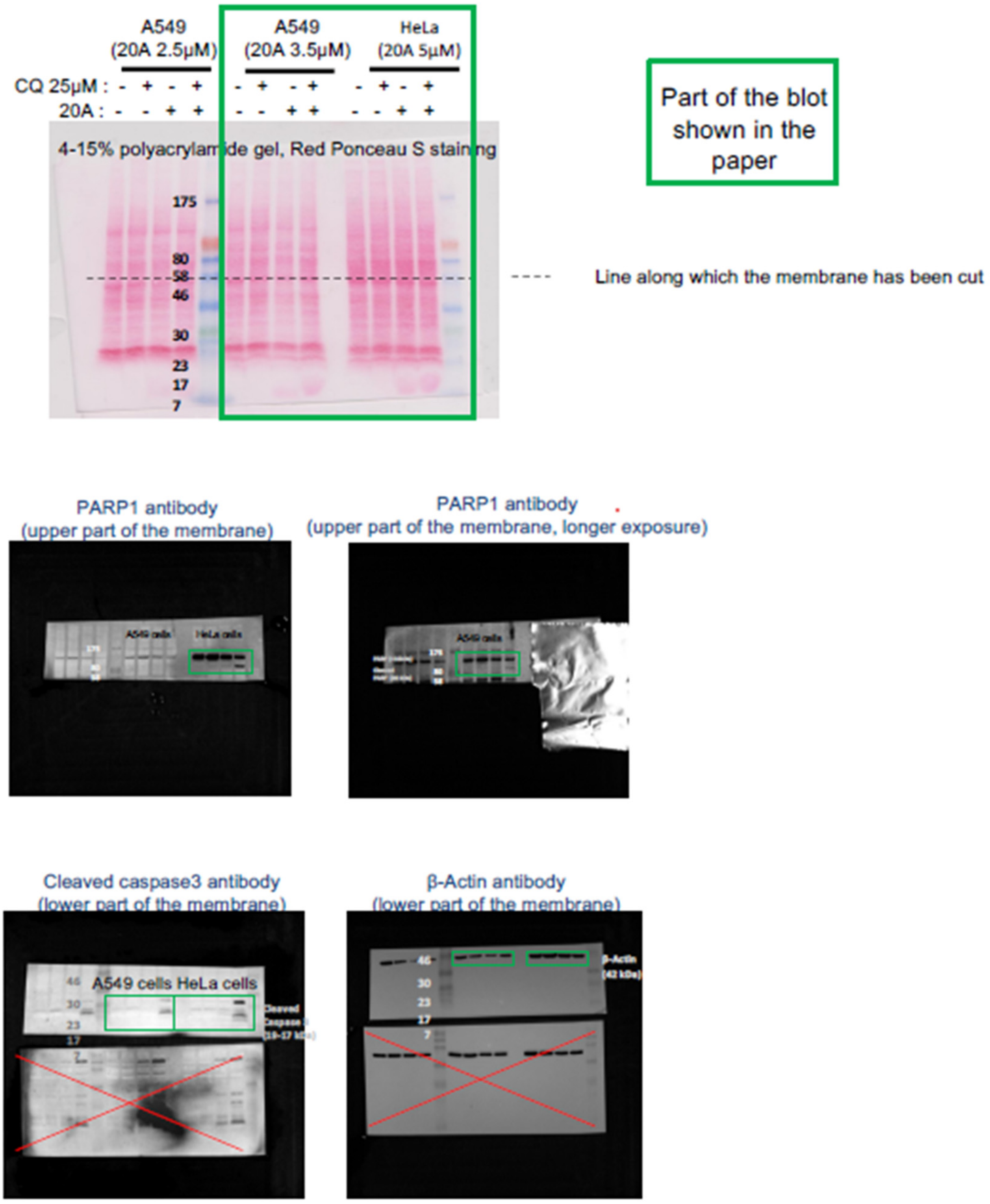

Figure S6. Full Western blot Images for PARP1 and cleaved forms of caspase 3. presented in Figure 5. Densitometry analyses are presented in Figure 5.

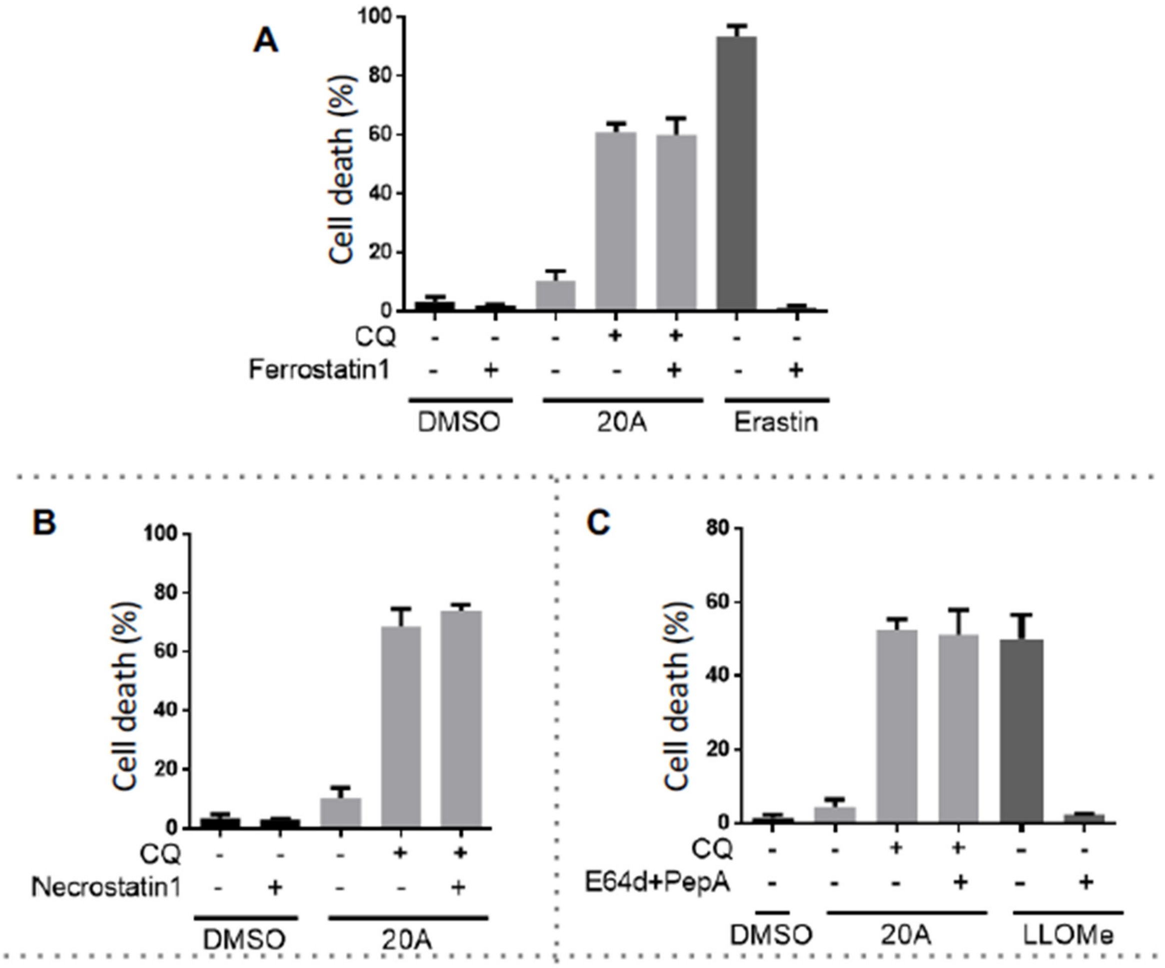

Figure S7: Evaluation of involvement of ferroptosis, necroptosis and cathepsin activity in cell treated 20A plus chloroquine. (**A**) A549 cells were treated or not with 5 µM Ferrostatin 1 two hours prior to the addition of either 20A (3.5 µM) or Erastin (10 µM). Where indicated, cells are also exposed to 25 µM chloroquine. Cell death was assessed by measurement of propidium iodide uptake. The data represents the mean ± SD of 3 values obtained from one experiment. (**B**) A549 cells were treated or not with 60 µM Necrostatin 1 two hours prior to the addition of 20A (3.5 µM) for 24 h. Where indicated, cells are also exposed to 25 µM chloroquine. Cell death was assessed by measurement of propidium iodide uptake. The data represents the mean ± SD of 3 values obtained from one experiment (**C**) A549 cells were treated or not with E64d (30 µM) + Pepstatin A (30 µM) two hours prior to the addition of either 20A (3.5 µM) or 4 µM LLOMe. Where indicated, cells are also exposed to 25 µM chloroquine. Cell death was assessed by measurement of PI uptake. The data represents the mean ± SD of 3 values obtained from one experiments.

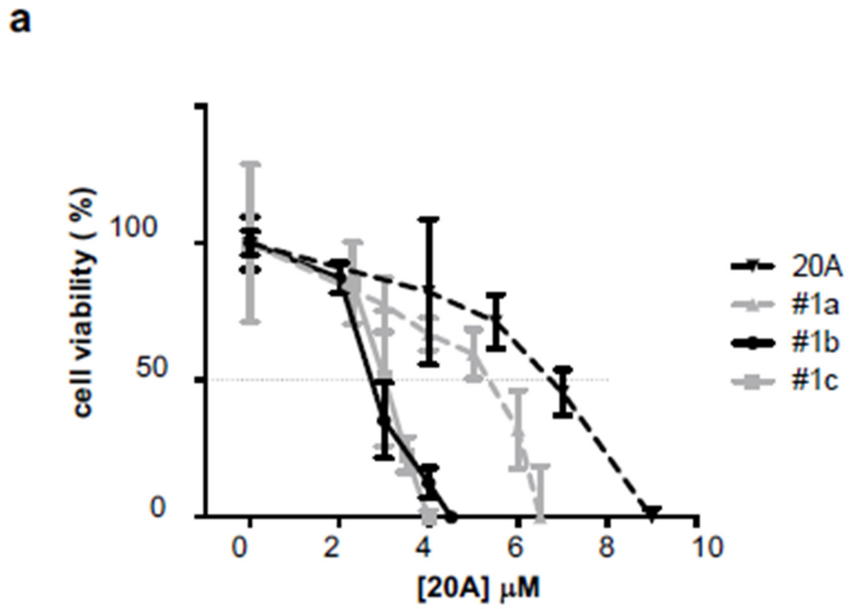

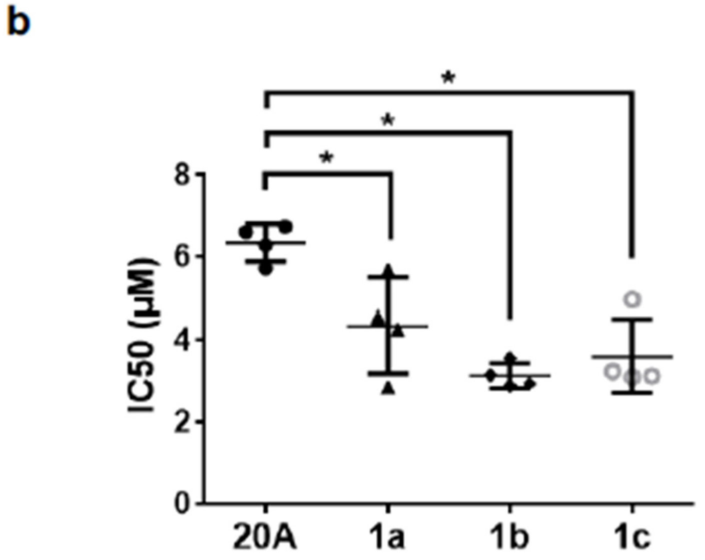

Figure S8. Cell viability assay for 20A derivative compounds (**a**) HeLa cells were treated with concentrations of 20A, 1a, 1b and 1c G4 ligands ranging from 1 to 10 μM for 24 h. Cell viability was assessed by the MTT assay. Plotted are mean ± SD of one representative experiment performed in quadruplicate. (**b**) IC50 is scored for each ligand (Mean ± SD, 4 independent experiments).

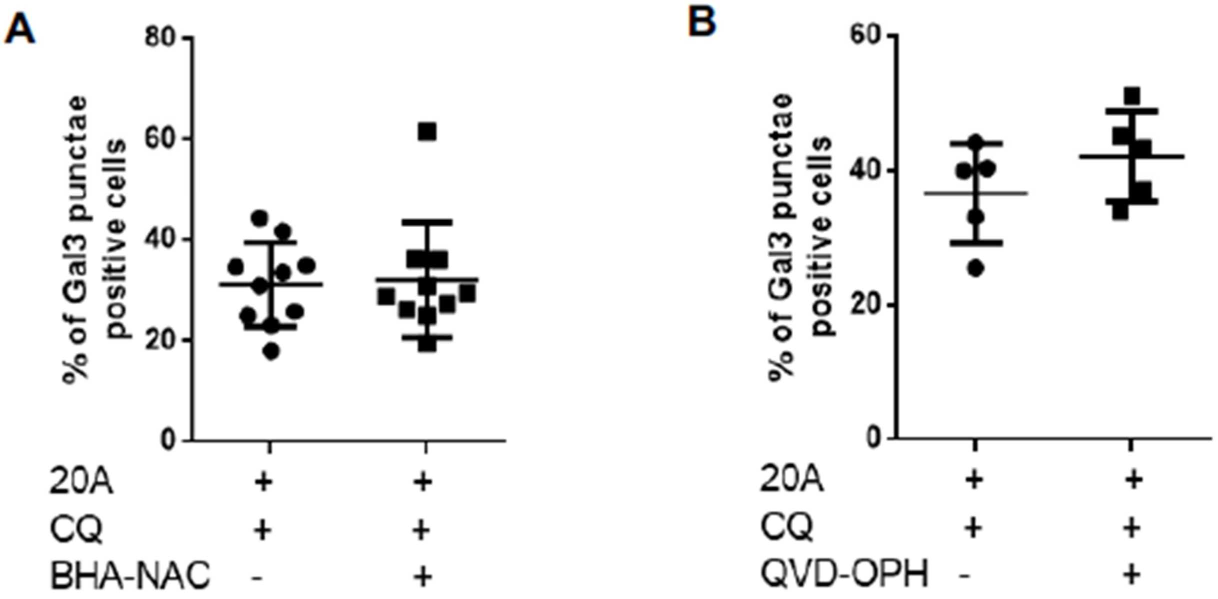

Figure S9. Role of caspase and ROS in LMP induced by 20A plus Chloroquine. (**A**) Galectin3-mcherry expressing U2OS cells were pre-treated for 2 h with or without 100 μM BHA and 5 mM NAC, followed by 24 h treatment with a combination of 3 μM 20A and 25 μM chloroquine. The percentage of cells displaying at least one Galectin3 puncta was quantified. Data are presented as mean ± SD of 10 values obtained from 10 randomly chosen areas in each of the two independent experiments. (**B**) Galectin3-mcherry expressing U2OS cells were pre-treated for 2 h with or without 20 μM QV-D-Oph, followed by 24 h treatment with a combination of 3 μM 20A and 25 μM chloroquine.The percentage of cells displaying at least one Galectin3 puncta was quantified. Data are presented as mean ± SD of 5 values obtained from 5 randomly chosen areas in one experiment.

Figure 2a : full picture for the Western blot ATG7

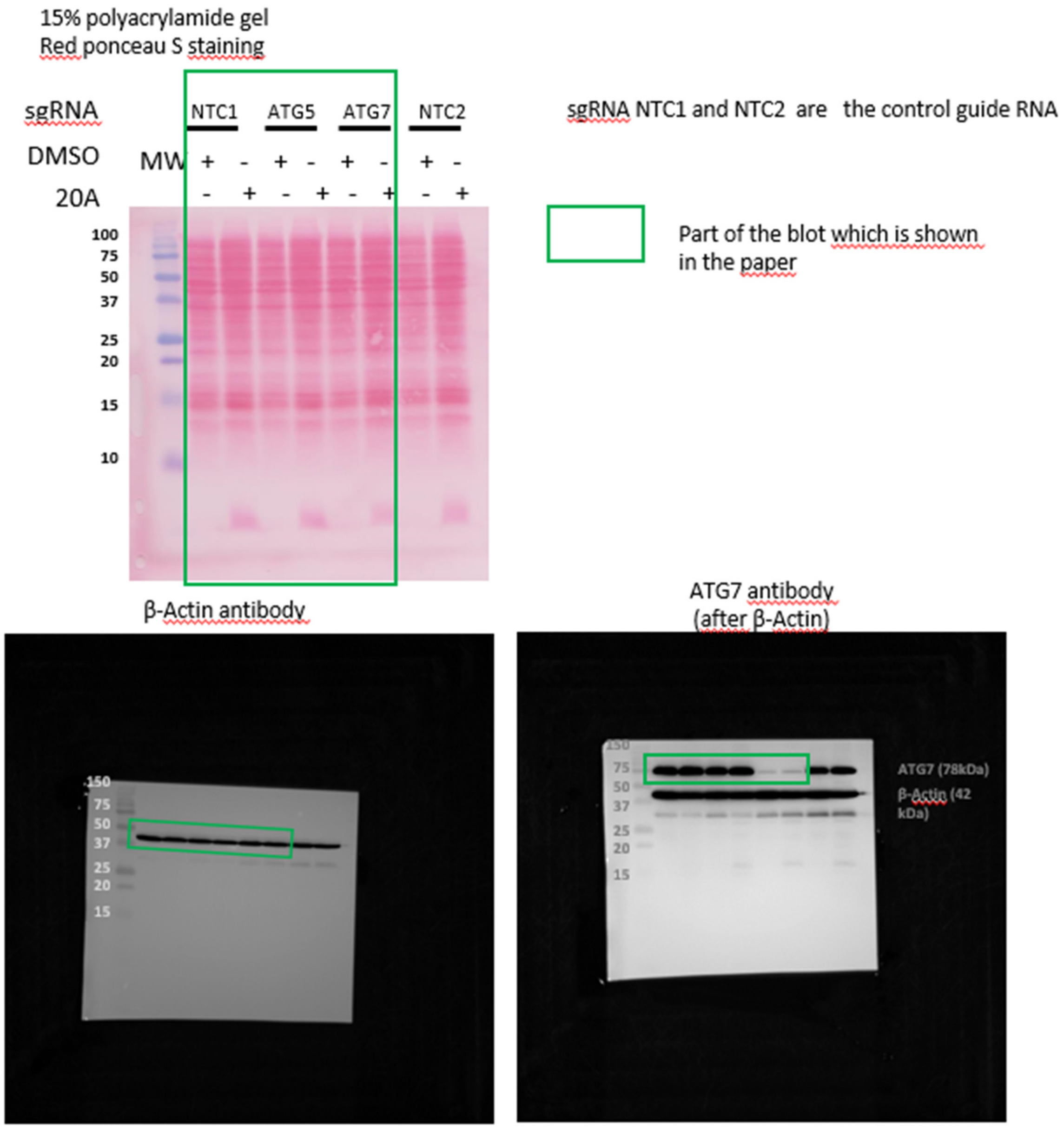

Figure 2 : full picture for the Western blot ATG5

12% polyacrylamide gel
Red ponceau S staining

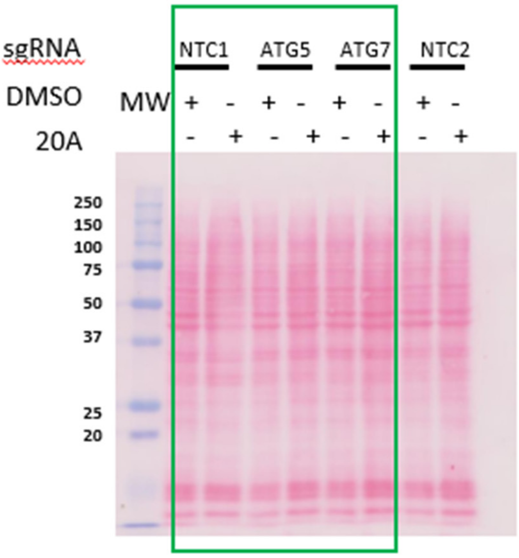

sgRNA NTC1 and NTC2 are 2 control guide RNA

Part of the blot which is shown in the paper

ATG5-ATG12 antibody

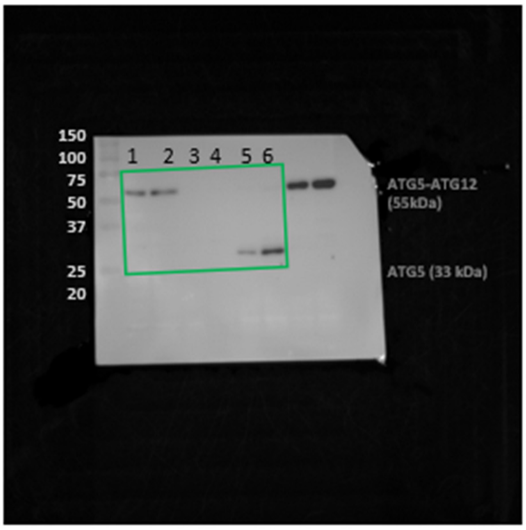

β-Actin antibody

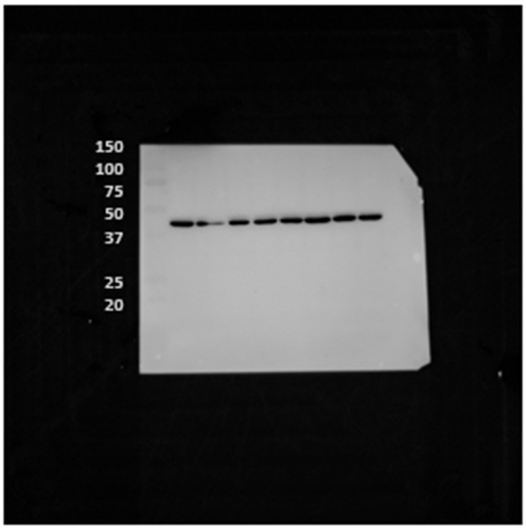

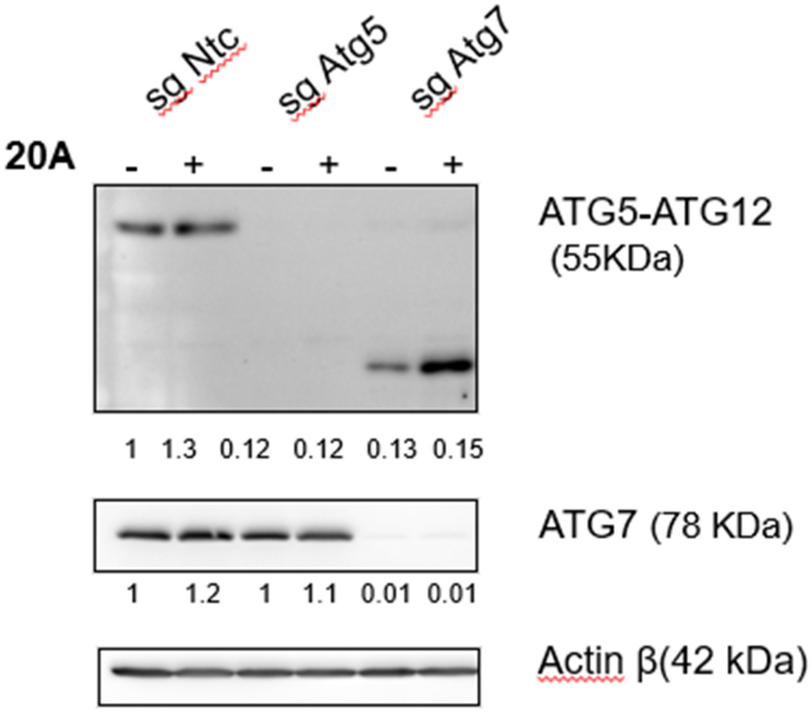

Figure S10. Detailed information about Figure 2a

Figure 4c : full picture for the Western blot PARP

Gradient 4-15% polyacrylamide gel
Red ponceau S staining

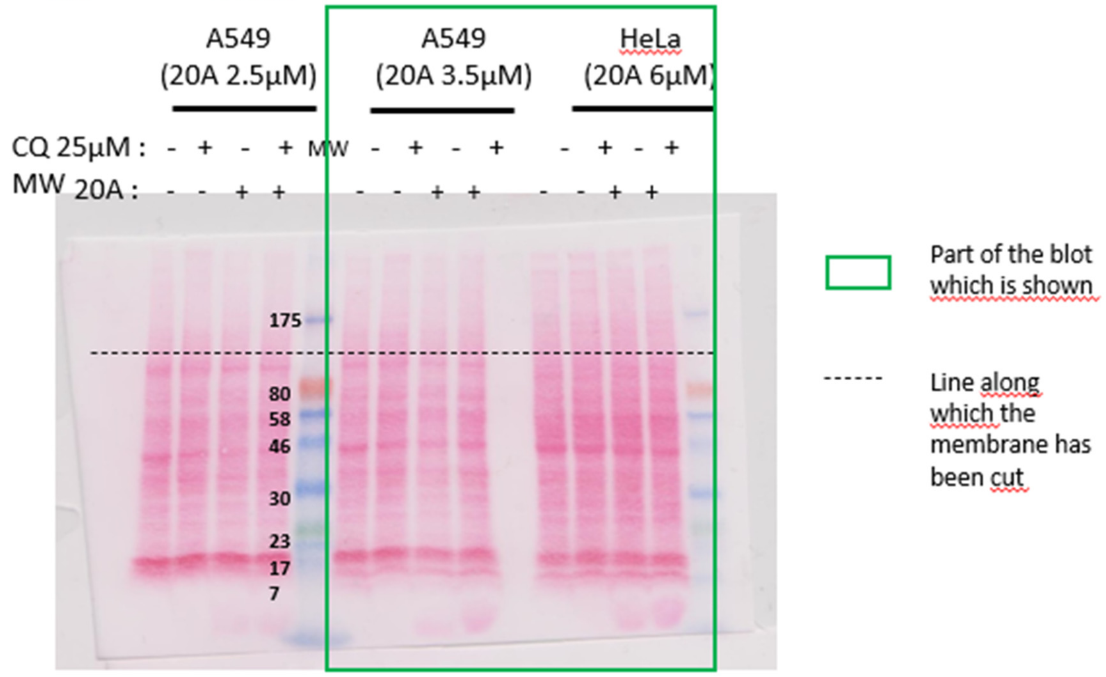

PARP antibody
(upper part of the membrane)

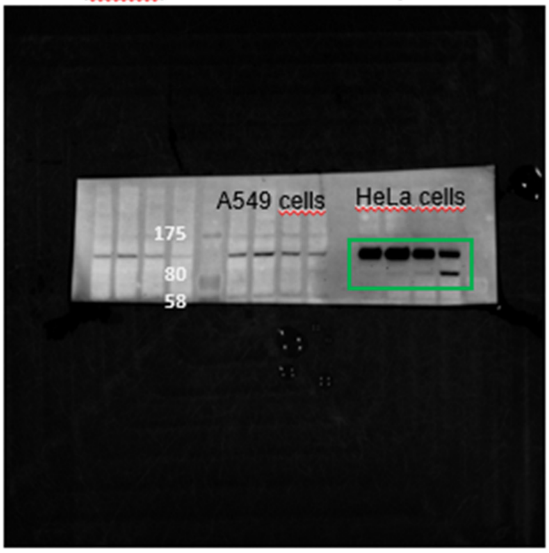

PARP antibody
(upper part of the membrane, longer exposure)

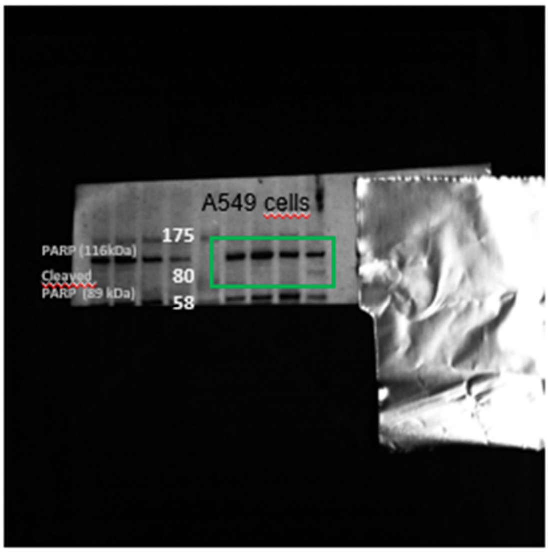

Figure 4 : full picture for the Western blot Cleaved Caspase 3

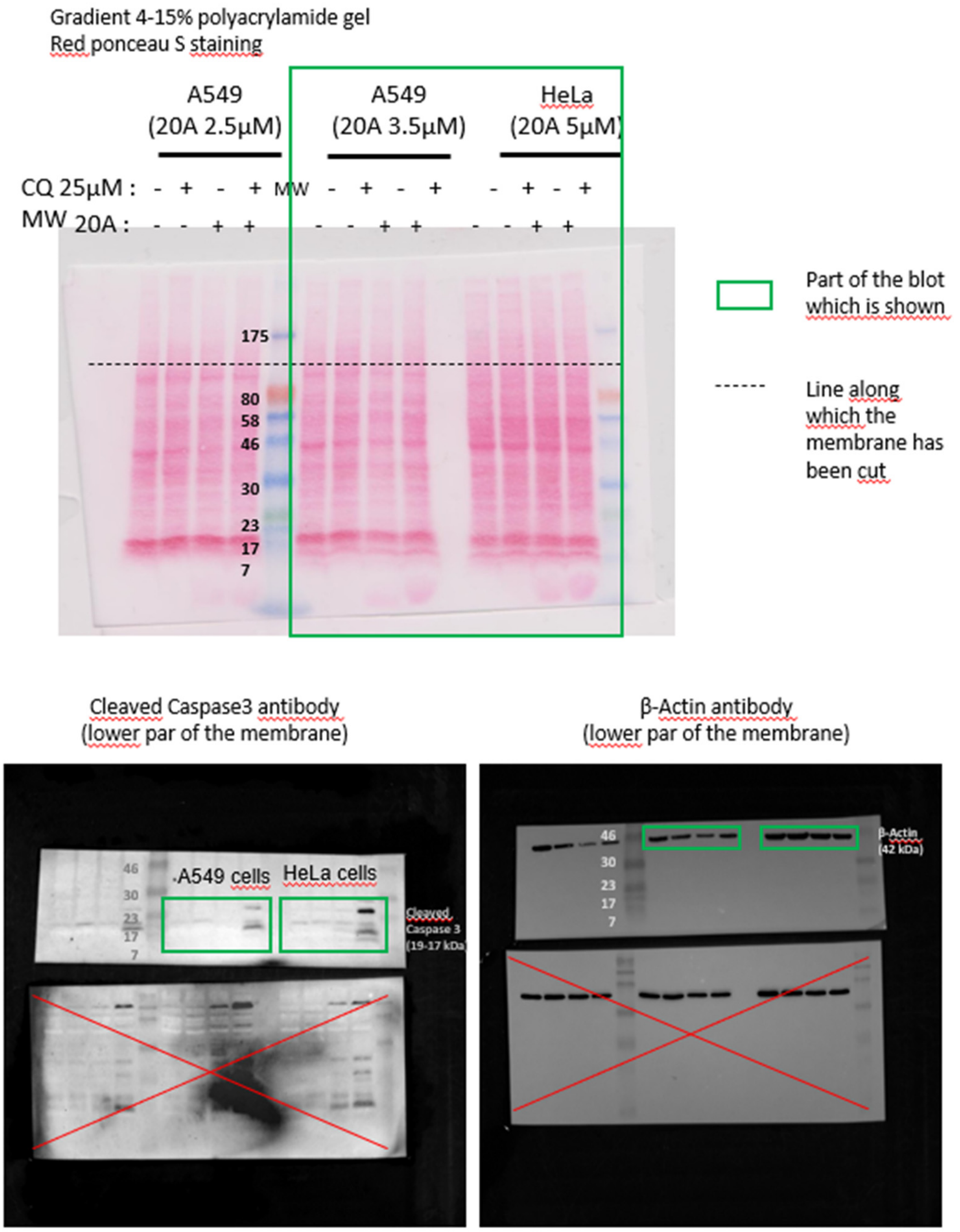

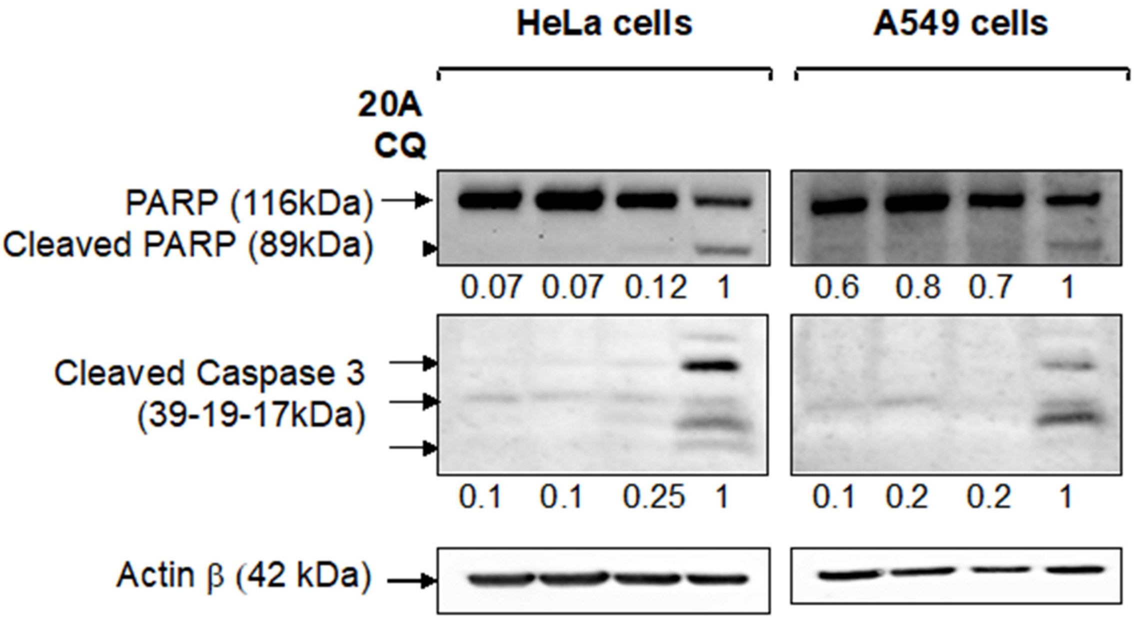

Figure S11. Detailed information about Figure 4c. The Western Blots were quantified and the results are expressed as ratio of cleaved caspased 3 forms/Actin β or cleaved Parp 1/Actin β.

© 2020 by the authors. Licensee MDPI, Basel, Switzerland. This article is an open access article distributed under the terms and conditions of the Creative Commons Attribution (CC BY) license (http://creativecommons.org/licenses/by/4.0/).
